# A Comparison of FES and SCS for Neuroplastic Recovery After SCI: Historical Perspectives and Future Directions

**DOI:** 10.3389/fneur.2020.00607

**Published:** 2020-06-30

**Authors:** Lynsey D. Duffell, Nicholas de Neufvillle Donaldson

**Affiliations:** ^1^Implanted Devices Group, University College London, London, United Kingdom; ^2^Aspire CREATe, University College London, London, United Kingdom

**Keywords:** FES, SCS, neuroplasticity, spinal cord injury, FES-cycling, locomotor training, neuroprosthesis, posterior nerve roots

## Abstract

There is increasing evidence that neuroplastic changes can occur even years after spinal cord injury, leading to reduced disability and better health which should reduce the cost of healthcare. In motor-incomplete spinal cord injury, recovery of leg function may occur if repetitive training causes afferent input to the lumbar spinal cord. The afferent input may be due to activity-based therapy without electrical stimulation but we present evidence that it is faster with electrical stimulation. This may be spinal cord stimulation or peripheral nerve stimulation. Recovery is faster if the stimulation is phasic and that the patient is trying to use their legs during the training. All the published studies are small, so all conclusions are provisional, but it appears that patients with more disability (AIS A and B) may need to continue using stimulation and for them, an implanted stimulator is likely to be convenient. Patients with less disability (AIS C and D) may make useful recovery and improve their quality of life from a course of therapy. This might be locomotion therapy but we argue that cycling with electrical stimulation, which uses biofeedback to encourage descending drive, causes rapid recovery and might be used with little supervision at home, making it much less expensive. Such an electrical therapy followed by conventional physiotherapy might be affordable for the many people living with chronic SCI. To put this in perspective, we present some information about what treatments are funded in the UK and the US.

## Introduction

Life expectancy after thoracic Spinal Cord Injury (SCI) is now close to normal in the western world (1). Around the time of the Second World War, the expectation was that there could be no recovery from SCI after the acute period: Cajal (2) had stated that neurons in the spinal cord were incapable of regeneration after injury. However, the Sygen study, carried out in the 1990s, showed that substantial spontaneous recovery was occurring in the months after injury (3): in people with incomplete injuries, motor scores reached a plateau 16 weeks after injury.

Sherwood et al. (4) showed, by neurophysiological tests, that 74/88 (84%) of patients diagnosed as having “complete” injuries, based on clinical tests, in fact retained some functional neurons passing the lesion; they proposed to describe these patients as *discomplete*. These spared neurons provide an opportunity for long-term recovery in people living with chronic incomplete and discomplete SCI. Indeed, Bareyre et al. (5) showed, in rats with a hemisection of the cord, that corticospinal neurons on the lesion side could sprout to make functional connections below the lesion. Friedli et al. (6) found, in 400 patients with tetraplegia, that asymmetry in the lesion allowed greater recovery, and also that, in monkeys, recovery correlated with corticospinal “detour circuits” below the injury. Many animal studies have demonstrated recovery after SCI following treadmill training, and this has reinforced the view that *Activity-Based Therapy* may enable patients with incomplete lesions to walk (7). Hubli and Dietz (8) described the knowledge gained as a “*physiological basis for neurorehabilitation*: a sufficient combination of sensory cues is required to generate and improve locomotor patterns.”

Many methods for rehabilitation have been tested, however, it is still far from clear what is most effective, or even what exactly the goal of the rehabilitation should be. This paper provides a historic review of rehabilitative interventions that incorporate electrical stimulation (ES), targeting lower limb peripheral nerves or nerve roots, combined with activity-based rehabilitation. Differences in approach are considered, in terms of electrode location, stimulation parameters (pattern and intensity), activity-based rehabilitation type and descending drive. We attempt to identify which factors might be most influential in achieving functional recovery; to that end, the review is focused on studies that incorporate International Standards for Neurological Classification of SCI (ISNCSCI) motor scores as an outcome measure. How, in the future, these interventions may be used more widely both for those with motor complete [ASIA Impairment Scale (AIS) A and B] and motor incomplete (AIS C and D) SCI, is then discussed.

Normal terminology used with ES is introduced in Table 1.

**Table 1:** Terms used in this field are often inexact, imprecise or even misleading. In this review, we are using the customary terms but offer this table in explanation.

FES (Functional Electrical Stimulation)	*Functional* originally meant that the stimulation restored normal function to paralyzed muscles. Its general meaning is unclear. We use it to mean stimulation of peripheral nerves with the primary purpose of causing muscle contractions.
SCS (Spinal Cord Stimulation)	Stimulation, primarily of the posterior (sensory) nerve roots.
Epidural SCS	Stimulation using an electrode array facing the dura in the posterior spinal canal.
Transcutaneous SCS	Stimulation using one or more electrodes on the skin in the lumbar region. The current can stimulate the posterior roots but also, to some extent, the cutaneous afferents and the trunk muscles.
Neuromodulation	May be by chemical or electrical methods. Tonic electrical stimulation is usually applied by an Implanted Pulse Generator (IPG). An example is Deep Brain Stimulation.
Neuroprosthesis	Used to describe devices that have a more complicated interaction with the nervous system than neuromodulators, stimulation being applied conditionally, following some physiological input, or closed-loop. Examples are foot-drop stimulators (see text) or demand cardiac pacemakers.

### Functional Electrical Stimulation (FES)

*Functional Electrical Stimulation* (FES) describes stimulation through electrodes placed transcutaneously over peripheral nerves or muscle bellies, which activate mixed peripheral nerves as they branch into the muscle. FES may also describe stimulation through implanted electrodes placed on ventral nerve roots. FES after SCI has been applied in many clinical and experimental settings since the early 1970s. Primarily, lower-limb FES has been used to achieve walking and cycling; these activities form the focus of this review.

#### FES-Walking

Liberson et al. (9) described a neuroprosthesis for correcting foot-drop in 1961. Patients with hemiplegia due to lesions in the brain or spinal cord caused by SCI, stroke, multiple sclerosis, and other conditions, had a device which stimulated the peroneal nerve during the swing phase of gait, lifting the foot clear of the ground. It took several decades before this treatment became established in clinical practice, but it is now broadly available. The device may use electrodes fixed on the skin over the nerve or implanted; the latter is helpful for people who are allergic to the self-adhesive electrodes, or who find it difficult to position the electrodes with sufficient accuracy.

When these neuroprostheses were developed, stimulation of the motor neurons was the aim, however, another important effect was inhibition of the plantarflexors (10); foot-drop often being caused by spasticity in these strong antagonists. Another discovery was a so-called *carry-over effect* (11). Patients with stroke who had been using the device for some time found that it was no longer necessary. Using it had caused sufficient recovery for them to be able to walk without the device. For them, a neuroprosthesis had acted as a therapy.

Between about 1975 and 2000, an active goal of research was to develop motor neuroprostheses to enable paraplegics with complete spinal cord lesions to walk during activities of daily living. Because most of the leg muscles in these patients will contract if stimulated, it seemed reasonable that if enough channels of stimulation were available, crude walking should be possible. Groups in Vienna (12, 13), London (14–17), and Cleveland (18) developed multi-channel stimulators and implanted them into volunteers. The results were disappointing; no really practical prosthesis was demonstrated. The three main reasons for lack of success were the following.

- Rapid muscle fatigue. The force produced by electrically-stimulated muscle declines rapidly, limiting the endurance for activities of daily living. This decline is usually attributed to the *inverse recruitment* of the stimulated nerve: because the larger axons are stimulated first, as stimulus intensity increases, fast muscle fibers are activated before slow fibers, which is opposite to the normal order (19). However, this explanation is insufficient. When SCI people are trained enough, their muscles can be converted largely to more oxidative fiber-types, and yet they still fatigue rapidly (20, 21).
- There is no satisfactory site for electrodes. Electrodes could be: (a) self-adhesive electrodes on the skin over the target muscles; (b) the tips of percutaneous wires that are inserted into the muscles through the skin (22); (c) electrodes, usually in an insulating cuff, surgically-implanted on the nerve; or (d) electrodes on the anterior nerve roots in the spinal canal. The disadvantages of each are: (a) too tedious to don and doff the electrodes every day, and not selective enough; (b) too dangerous due to infections and broken wires; (c) requires very extensive surgery with associate risk and poor reliability; (d) not possible to get enough different combinations of muscles for several useful functions (16).
- Little progress was made with the user interface. An early concept was that motor signals would be taken from the motor cortex and used to drive the neuroprosthesis. For lower limb rehabilitation, this does not seem ethically justifiable and, in practice, when stepping was controlled by the patient, it was usually by manually-operated switches on whatever was held to provide balance (usually a walking frame) (23). For the very crude stepping that was achieved, switches were adequate, but perhaps they would not do outside the clinic when confronted with curbs, bends or rough ground. For outdoor walking, some method that gives greater range of control, would be necessary, and this should ideally be innate.

At the same time, locomotor treadmill training (LTT), incorporating body-weight-support and therapist- or robotic-assistive step training, had become popular. Supported LTT was thought to improve function through the responsiveness of *central pattern generators* (CPG) to afferent stimuli noted in mammalian quadrupeds (24, 25). In people with chronic SCI, improvements in walking function (26–30) and muscle properties (31, 32) were reported after LTT, however a systematic review conducted in 2013 concluded that evidence on the effectiveness of locomotor therapy was limited (33). Few LTT studies have reported changes in ISNCSCI motor scores. One recent study did report motor scores from 60 participants with motor incomplete SCI (0.1–45 years post-injury) after 120 sessions of body-weight supported LTT (34). The training time averaged 11 months (sessions every 3 days), and the mean improvement in ISNCSCI motor scores was 6 points. LTT has also been combined with FES, and improvements in gait parameters were found to be slightly better than LTT combined with manual assistance (33). Field-Fote and Roach (30) reported improvements in walking function and lower extremity motor scores following 12 weeks of LTT combined with FES, training 5 days/week, but the improvements were not superior to LTT with manual or robotic assistance, or overground walking training without FES. Possover (35) used an implanted neuroprosthesis (electrodes were placed on the pelvic, sacral and femoral nerves) to provide FES during locomotor training in 4 people with SCI (AIS A—C). Impressive improvements in ISNCSCI motor scores were noted following 12 months of training (mean 12.8 points), and three participants became capable of voluntary weight-bearing standing and walking a few meters with a walker without FES (35).

#### FES-Cycling

Enabling people to cycle on a recumbent tricycle or exercise ergometer is much simpler than FES-walking and supported-LTT. The feet are fixed to the pedals, constraining their motion, and the moving mass of the mobile tricycle or flywheel in the ergometer smoothens the movement. In the 1980s, Petrofsky et al. (36) first described the development of a “computerized, closed loop control stimulator, and cycle ergometer for people with SCI.” Each of the main muscle groups (e.g., knee extensors, hip flexors, etc.) should be stimulated for a range of crank angle over which the muscles contribute positive propulsion, which is easy to understand and implement. Another advantage is that, while seated with the machine, falling is not likely.

Many FES cycling studies were subsequently carried out, where the purpose was to use the large paralyzed muscles as healthy exercise: benefits to muscle properties are now well-established (20, 37–51), and cardiopulmonary benefits have also been reported (40, 44, 45, 52–57). Based on these findings FES has become standard of care after SCI in clinical settings; cycle ergometers are mostly used, which have a motor to establish a cadence and the FES reinforces that motion unless there is spasticity. These studies typically recruited people with complete injuries only, to avoid discomfort caused by mixed peripheral nerve stimulation in people with incomplete SCI. At the time it was believed that motor recovery was unattainable in people with complete injuries, therefore it was seldom measured or reported.

In 2000 we reported a case study of an individual with incomplete SCI, who had been neurologically stable for 10 years before he started FES-cycling, averaging 22 min per day (58). His tricycle was usually on rollers indoors but sometimes he would cycle outdoors, and he increased his rides up to a maximum of 12 km. We discovered that he had then recovered some control over a previously completely paralyzed knee. He said that this improvement enabled him to move round in his kitchen with only one stick, instead of two, and he became able to pick objects up from the floor while standing. Needless to say, these gains were not rehabilitation targets but they were significant improvements to his quality of life.

Soon after, McDonald et al. (59) reported a case study on an individual with motor complete SCI (AIS A) who initiated a program of “activity-based rehabilitation,” consisting of FES cycling supplemented with ES of abdominal and upper limb muscles, 5 years post-injury. Over 3 years of therapy, he recovered substantial motor function (ISNCSCI motor scores improved from 0/100 to 20/100), and his AIS Grade improved from A to C. The same group later reported motor recovery in a group of 25 people with chronic complete and incomplete SCI (17 were AIS A) who carried out FES cycling 3 times/week for an average of 30 months (60). Their ISNCSCI motor scores improved by an average of 8.0 points, compared with an average change of 0.6 points in the control group (*n* = 20) who only received range of motion and stretching exercises.

Following these discoveries, we developed an ergometer for FES-cycling with virtual reality (VR) to encourage volitional effort, the *iCycle*. Its unique feature is that there is biofeedback to the VR so that the speed of the avatar depends on the real crankshaft torque when there is no torque due to stimulation. In a small clinical trial, we noted an improvement in ISNCSCI motor scores in people with chronic, incomplete SCI (AIS C and D) after 1 month of training on the iCycle, training 3 times/week. The mean improvement was 1.5 points after training, and 4.7 points after follow up (1 month later) (61).

**Figure 1:**
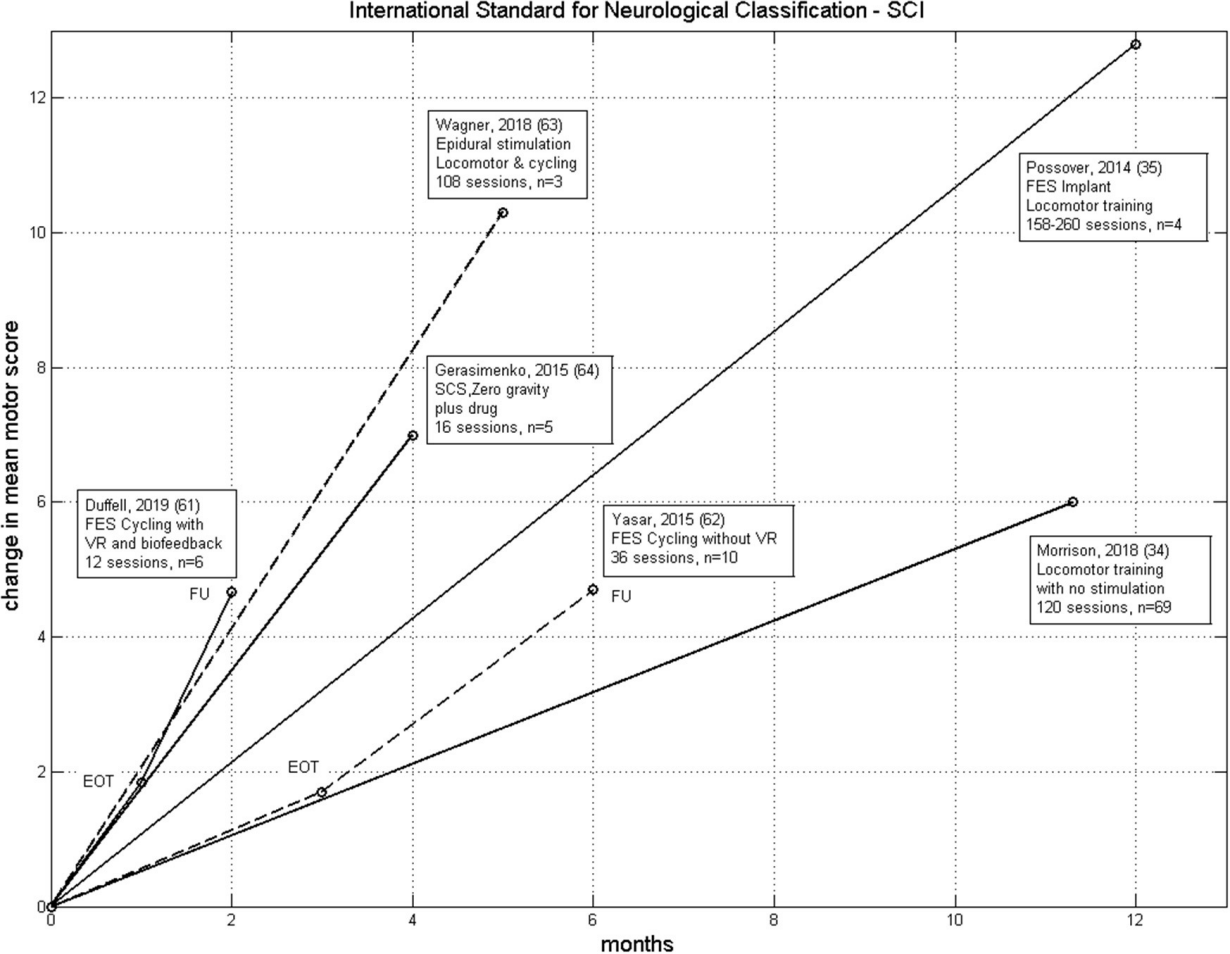
Comparison of the rate of change of ISNCSCI motor score for six therapies from Table 2. These have *n* > 1 and non-negligible improvement in motor score. We excluded Sadowsky et al. (60) because of the very wide range of training in that study. LT without electrical stimulation (34), FES-Cycling (62), FES-Cycling with virtual reality and biofeedback (61), SCS Neuroprosthesis (63), FES Neuroprosthesis (35) and SCS with drug and zero-gravity training (64). EOT, End of Therapy; FU, Follow-Up.

Our data can be compared with the data presented by Yaşar et al. (62) from a trial on 10 people, also with chronic incomplete SCI (AIS C and D). FES cycling was carried out 3 times/week for 3 months with follow-up 3 months later. In that study, the participants were instructed not to contribute to the cycling voluntarily, but just allow the stimulation to occur. The mean change in ISNCSCI motor score at the end of therapy and follow-up were nearly equal to our iCycle data, except the rate of improvement was three times greater with the iCycle (Figure 1). This supports our idea that voluntary drive, encouraged by the virtual reality and biofeedback, does promote recovery. However, our data is equivocal, we found no correlation between the change in motor score and dose (time spent cycling).

Passive cycling, without stimulation, has been investigated as a therapy and the literature recently reviewed by Phadke et al. (65). There are cardiovascular, musculoskeletal and neurological effects, the latter being changes to the H-reflex and spasticity, but none of the studies measured neurological outcome after multiple sessions to see whether there were changes in ISNCSCI motor score.

**Table 2:** Treatment of SCI patients with chronic injuries by electrical stimulation: change in ISNCSCI Motor Score.

**Paper**	***n***	**Motor complete/ incomplete**	**SCS/FES**	**Implant/ surface**	**Tonic/ phasic**	**Walking/ cycling**	**Therapy Duration (months)**	**Therapy intensity (sessions/week)**	**Total number of training sessions**	**Mean (range): improvement in ISNCSCI Motor Score**
Possover (35)^a^	4	Complete/incomplete (B/C)	FES	Implant	Phasic	Walking	12	3–5	156–260	12.8 (1–21)
McDonald et al. (59)^b^	1	Complete (A)	FES	Surface	Phasic	Cycling	36	7	1,092	20
Wagner et al. (63)	3	Incomplete (C/D)	SCS	Implant	Phasic	Walking + cycling	5	5	108	10.3 (4–16)
Sadowsky et al. (60)	25	Complete/incomplete (A/B/C)	FES	Surface	Phasic	Cycling	29.5 (Range 3–168)	3	360 (Range 36–2016)	8.1 (NR)
Gerasimenko et al. (64)	5	Complete (B)	SCS + drug	Surface	Tonic	Walking (zero-gravity)	4	1	16	7 (NR)
Yaşar et al. (62)	10	Incomplete (C/D)	FES	Surface	Phasic	Cycling	3	3	36	4.7 (NR)^*^
Duffell et al. (61)	6	Incomplete (C/D)	FES	Surface	Phasic	Cycling	1	3	12	4.7 (0–13)^*^
Angeli et al. (89)	4	Complete (A/B)	SCS	Implant	Tonic	Walking	Range 6–20	7	168–560	0.25 (0–1)
Carhart et al. (88)	1	Incomplete (C)	SCS	Implant	Tonic	Walking	7	5	140	0
Grahn et al. (85)	1	Complete (A)	SCS	Implant	Tonic	Walking	0.5	4	8	0

a *Possover applied continuous low-frequency pelvic nerve stimulation between the training phases*.

b *McDonald supplemented FES cycling with continuous stimulation of trunk muscles*.

* *Improvement in ISNCSCI motor score taken from follow-up data, as opposed to end of training data*.

*NR, Not Reported*.

### Spinal Cord Stimulation (SCS)

*Spinal Cord Stimulation* (SCS) describes stimulation through electrodes implanted in the epidural space inside the spinal canal or placed transcutaneously over the spine. Epidural SCS was first tested in 1967 by Shealy et al. (66), who described it as *dorsal column stimulation*, as a way to alleviate chronic pain. It is now well-established as a clinical treatment for pain using chronic implanted stimulators (67). Its effects on the motor system were noticed in the 1970s, notably reduction in spasticity and partial recovery. Its history has been reviewed by Minassian et al. (68). Briefly, in 1998, Dimitrievic et al. (69) showed that SCS can excite the CPG in humans after SCI; the neural circuits within the lumbar cord which give coordinated contractions of the leg muscles appropriate for stepping. Low-intensity stimulation, below a level necessary to produce any such motor response in the paralyzed muscles, may enable a discomplete patient to produce EMG responses by volition. Dimitrievic proposed that this offered a therapeutic pathway for SCI in 1984 (70). The mechanisms of recovery have been reviewed more recently by Taccola et al. (71).

The terms *dorsal column stimulation* and *spinal cord stimulation* may actually be inaccurate. While, within the chronic pain literature, SCS is widely considered to activate dorsal column fibers both orthodromically and antidromically [for review see (72)], others have shown that, with electrodes on the dura or even on the back, the neurons that are first stimulated within the spine are the large diameter sensory fibers of the posterior roots (73–75). These include A-alpha fibers conducting proprioceptive feedback from the muscle spindle afferents. Most likely, the activation of multiple structures, such as dorsal column fibers, the dorsal horn and posterior/ventral roots, contribute to the overall effects of SCS. In the studies reviewed here, the peripheral nerves (posterior roots) are targeted, and the susceptibility of evoked potentials to homosynaptic depression is typically used to verify posterior root activation (76, 77).

The activation of CPGs by non-invasive tonic SCS has been demonstrated in able-bodied people (78, 79). In these experiments, the limbs were suspended to remove afferent input, and the participants are asked not to voluntarily contribute to the movement, to minimize supraspinal influence. Locomotor-like behavior in response to the tonic SCS occurred in only 7 out of 65 participants tested (79); supraspinally mediated inhibition of the spinal circuitry may have prevented activation of CPGs in the majority of able-bodied participants. In people with SCI, where descending input has been compromised, CPGs have been activated more robustly with both non-invasive (64) and invasive (69) tonic SCS. There are also several observations that tonic invasive and non-invasive SCS enables descending motor control; people with chronic SCI were able to generate voluntarily-driven movements and step-like activity in the presence of SCS, which were otherwise not possible (64, 80–88).

Carhart et al. (88) tested the use of an implanted stimulator for SCS in one volunteer with incomplete cervical injury who trained with *tonic* stimulation on a treadmill with partial body-weight support. He trained five times per week for 150 sessions and changed from being a wheelchair-user to being a community walker, however his ISNCSCI motor Score remained 15/50. Angeli et al. (86, 89) reported a similar method, stimulation being applied *tonically* to four patients with chronic injuries, two AIS A and two AIS B. The first of these patients had been described in an earlier case study paper (82). All four had 40–45 sessions lasting 2 h of treadmill training before implantation. The stimulation through the 16-electrode array was optimized and then used chronically. After implantation, they trained once or twice per day, walking on the treadmill, walking over ground, or standing. While the stimulator was on, the AIS A's became able to stand with support while the AIS B's could also walk over ground. The AIS B's took 278 and 81 training sessions, respectively to reach this outcome. In only one of the four did the motor score change as a result of this training: one of the AIS B's changed from 0/50 to 1/50, the other remained 0/50. This method may be described as *neuromodulation* because tonic stimulation is only exciting the spinal cord while control is from the patient's brain (at least when the therapists no longer intervened). This is an intriguing result because the functional improvements were substantial with the stimulator on, despite almost no change in motor score.

Wagner et al. (63) used a neuroprosthetic approach with SCS to rehabilitate three patients (AIS C or D) for walking. They used similar epidural electrode arrays over the lumbar cord but used “spatio-temporal stimulation.” For each leg, the gait cycle was divided into three phases in which different motor neuron pools were targeted. In each phase the combination of electrodes, stimulation currents and frequencies were optimized. All three patients could take steps as soon as this epidural stimulation was started. Their therapy lasted 5 months, five sessions per week after which 2/3 could do overground walking with handles for balance. They had all gained some ability to move their legs without the stimulation and their ISNCSCI motor scores had increased by 16, 11, and 4 points. When walking outside, they wore accelerometers on their shoes for phasing and a belt-worn controller that could be commanded by voice.

The studies discussed in this review, which incorporated INSCSCI motor scores as an outcome measure, have been summarized in Table 2. All the results are from chronically-injured people. The papers have been ranked by change in INSCSCI motor score. Figure 1 shows the rate of change of motor score for some of these studies.

## Discussion

In this review, we have described the rehabilitative interventions, incorporating electrical stimulation (ES) targeting lower limb peripheral nerves, that have evolved since the 1960s for people living with SCI. We have used the ISNCSCI motor scores as a primary outcome measure for neuroplastic effects because it is a clinically-relevant and a standardized measure of motor function after SCI. Changes in ISNCSCI motor scores represent therapeutic, neuroplastic effects of an ES-based intervention because ES is not used during the assessment. There is currently no consensus on a *minimally important difference* (MID) in ISNCSCI motor score, which could be considered as a clinically relevant improvement. Nevertheless, in Table 2, we have ranked the papers by change in INSCSCI motor score. Based on this table, the importance of the training dose, and the type of stimulation and training used, for optimal neuroplastic effects, will be discussed. A summary of the advantages and disadvantages of each technique is provided in Table 3. Finally we discuss costs to put the science in a healthcare context.

**Table 3:**
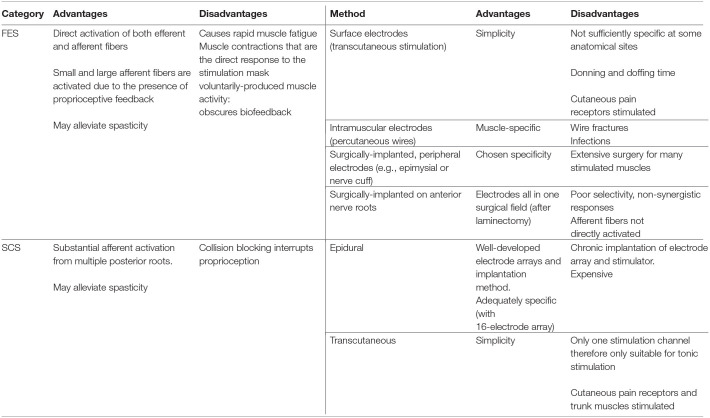
A summary of the advantages and disadvantages of Functional Electrical Stimulation (FES) and Spinal Cord Stimulation (SCS) and the available methods.

### Dose Effects

As shown in Table 2, the number of training sessions per week, and total number of training sessions completed, varied widely between studies. It is notable that the studies reporting the largest improvement in motor scores incorporated the highest frequency of sessions (5–7 sessions per week) and aggregate number of training sessions, pointing toward a dose effect. However, this does not hold true for the studies by Angeli (89) and Carhart (88), which reported no motor recovery despite a high dose of training; intensive LTT was provided without and then with SCS. While these studies reported remarkable improvements in standing and walking function in the presence of SCS, these improvements did not translate to improvements in motor scores.

A study by Harness et al. (90) explored dose effects in two groups of SCI participants: one experimental group (*n* = 21) who underwent “intense exercise” (actually six different exercises), and a control group (*n* = 8) who underwent self-regulated exercise, for 6 months. The control group exercised nearly twice as often, but the time per week was similar. The subjects in the experimental group gained in motor scores (by 5 points), whereas the control group did not show any gains. A dose effect was found in the experimental group only (*R* = 0.53; *p* < 0.02). They concluded that unstructured exercise might have less value for return of motor function in people with chronic SCI (90).

Clearly there is not a simple relationship between the dose of an intervention (session duration, weekly session frequency and total number of training sessions), and the outcome in terms of recovery of motor function. The stimulation parameters, and type of training carried out may have important affects in this relationship.

### Electrode Location

The key difference between FES and SCS is the anatomical sites where the electrodes are placed: close to the cord or close to the muscle. Despite this difference, both techniques activate large-to-medium diameter afferent fibers: afferent input is crucial to induce long-term plasticity, thus it is probable that similar mechanisms contribute to neuroplastic recovery with FES and SCS. Because SCS electrodes are often arranged so that all the lumbar roots can be stimulated, whereas FES electrodes are generally not applied to most lower limb muscles, it should be easier to directly activate more large afferents by SCS (75, 91, 92) than FES. However, there is an opposing effect which has been investigated by Formento et al. (92). Proprioceptive feedback (including the activation of smaller sensory fibers that are not usually targeted by the direct electrical stimulus), which is crucial in the control of human movement, may be substantially blocked during SCS. When axons are stimulated, action potentials propagate in both directions; in the large afferent fibers, the orthodromic action potentials travel to the synapses in the spinal cord while the antidromic travel toward the receptors in the periphery. If the antidromic action potentials meet naturally-generated orthodromic action potentials, they will annihilate each other—an effect called *collision blocking*. Because the largest afferent fibers are from the muscle spindles, this blocking causes loss of proprioception. The amount of loss depends on the proportion of these afferent fibers being stimulated, the stimulation frequency, and the position on the stimulating electrode along the nerve; the closer the electrode is to the spinal cord, the greater the blocking effect. Experiments showing this effect have been described by Formento et al. (93).

Loss of proprioception is not only a disadvantage for the patients' immediate motor control but may be fundamental to neuroplastic recovery. Takeoka (94) showed mutant mice, lacking muscle spindle afferents, could not recover from SCI like wild mice because the rearranged descending circuits were defective. It is thought that the monosynaptic connection between the spindle afferents and the motor neurons may be critical. This may go some way to explain why no changes in ISNCSCI motor score were achieved in the studies by Carhart (88) and Angeli (89) (Table 2), despite impressive functional recovery while stimulation was on. Given this disadvantage to SCS, Formento et al. (93) proposed a method of stimulation that mitigates the effect of collision blocking (use high frequencies at low amplitudes) that allows some proprioceptive feedback. However, setting up this stimulation is complicated and this difficulty may be an obstacle to widespread use because it will deter clinicians (95). The application of SCS *phasically* may, at least in part, overcome collision blocking.

The difference between electrodes being implanted or transcutaneous should also be considered, due to the possibility of activating different spinal and supraspinal structures with each technique. It can be noted from Table 2 that, among the studies applying phasic stimulation, those that used implanted electrodes achieved superior neuroplastic recovery compared with those using surface electrodes, which may be due to greater specificity being achieved with implanted compared to transcutaneous electrodes. The spread of stimulation during non-invasive FES causes activation of nearby, possibly antagonistic, muscles; this may hinder the intended functional activity. In addition, deep muscles cannot be activated by non-invasive FES. Implanted cuff electrodes placed on specific anterior nerve roots can be used to activate both deep and surface muscles, however it is still not selective enough, and there is a possible disadvantage of afferent fibers not being directly activated by the stimulus. With SCS, it has been shown that activation of the same afferent target neurons can be obtained with epidural and transcutaneous applications (75, 96), however, presumably greater specificity can still be achieved with epidural applications. In addition, transcutaneous SCS causes simultaneous activation of trunk muscles, which can be uncomfortable for people with retained sensation, particularly during tonic SCS; this limits the stimulation intensity that can be applied.

### Stimulation Parameters (Pattern and Intensity)

FES is routinely applied phasically, at an intensity sufficient to induce a motor response (i.e., supra-threshold). A single session of repetitive (phasic) supra-threshold stimulation (at 10–200 Hz), applied over a peripheral nerve, has been shown to transiently increase net corticospinal excitability (increased Motor Evoked Potential (MEP) amplitude) in both able-bodied people (97–100) and in people with incomplete SCI (101) due, at least in part, to reducing the activity of inhibitory cortical neurons (102, 103) [at a spinal level, FES has been shown to have either minimal or mainly inhibitory effects (104–107)]. A single session of *tonic, sub-threshold* peripheral nerve stimulation (known as TENS) has been shown to have the opposite effect, transiently reducing corticospinal excitability (108), with similar inhibitory effects at a spinal level (107, 109–111). Both FES (112) and TENS (113) have been found to be effective in the treatment of spasticity after SCI.

SCS is typically applied tonically rather than phasically, and the effects of tonic SCS on corticospinal excitability in humans has not been widely explored. One case report in a person with motor incomplete SCI observed increased corticospinal excitability following 14 sessions of tonic, low frequency (0.2 Hz) SCS applied in 10-min bursts of sub- and supra-threshold intensity (114). Although tonic SCS, applied at a supra-threshold intensity (at 15–50 Hz) initiates rhythmic CPG activity (69), tonic SCS is more often provided at sub-threshold intensities: at an intensity that enables voluntarily-driven movements in people with motor complete and incomplete SCI (64, 80–88). It is believed that this form of tonic SCS raises excitability in spinal circuitry, such that it becomes more responsive to descending input. However, in the pain management field, extensive animal research indicates that tonic, sub-threshold SCS induces or modifies the release of neuroactive substances involving GABAergic and cholinergic mechanisms, inhibiting hyperexcitability at the dorsal horn. Activation of supraspinal circuitry is also thought to contribute substantially to the attenuation of neural hyperexcitability via descending inhibitory tracts [for review see (72)]. SCS has also been reported to attenuate neural hyperexcitability and recover spinal inhibitory control in people with SCI (115–117), and has been successfully used in the treatment of spasticity (118, 119). The effects of tonic, subthreshold SCS on spinal hyperexcitability may be an important mechanism enabling voluntarily-driven movements in people with chronic SCI. Further work is required to elucidate the effects of sub-threshold SCS and FES, applied tonically or phasically, on spinal and corticospinal excitability.

Table 2 shows that both FES and SCS cause improvements in motor score, but neurological recovery (change in ISNCSCI scores) tends to be greater with phasic stimulation. It is difficult to compare the two recent (2018) SCS studies that used implanted electrodes; one applied tonic (89) and the other phasic (63) stimulation, but both appeared to be sub-threshold: at a level that enabled volitional control. The subjects in the study by Angeli et al. (89) were AIS A and B; they used tonic stimulation, showed little neurological recovery, but, with ES, could perform useful functions that were otherwise impossible. In contrast, the subjects in the study by Wagner et al. (63) were AIS C or D, their neuroprosthesis stimulated phasically, and rapid neurological change occurred allowing some new possible function without ES although function was better with ES. To what degree the greater recovery in the latter study is due to the phasic stimulation, and what is due to lesser initial paralysis, cannot yet be known. We await further results for each method with patients of all AIS grades.

The neuroprostheses described by Wagner et al. (63), which stimulates through epidural electrodes, can be compared to the implantable FES (motor) neuroprostheses from the 1980s. This new device solves all the three problems that beset those FES implants: movement is volitional, the leg movements being natural but reinforced by the stimulation; all the flexor and extensor motor neuron pools can be excited from this centrally-implanted electrode array; and finally, muscle fatigue should be slower because the motor neurons will be recruited in the natural order. The third point is, as far as we know, still only expectation based on normal physiology (120); actual rates of fatigue in SCI people have not been measured. Herman et al. (81) claimed in his case study that the patient's metabolism changed away from glycolysis with SCS, implying normal recruitment. Thus, the neuroprosthesis from Courtine's group (63) is a significant advance in neuroprosthetics. Making the system more convenient by implanting the accelerometers would not be a technical problem. It will be of great interest to see how well these devices work in people with motor complete SCI (AIS A and B); also, how well they are accepted, not just for gait training, but in ADL: will they actually be used instead of a wheelchair? What these studies do suggest is that after this much training, using the implants, the devices remain useful; their therapeutic effect has not been so great that they are no longer necessary, as was found in some 30% of patients with foot-drop (11).

### Activity-Based Rehabilitation Type

Locomotor Training has been popular for treating patients with SCI in recent years. One reason is probably that both patients and their specialist clinicians put functional walking high in a wish list after bowel and bladder control (121).

Another reason is the discoveries made from experiments with spinally-injured animals. The isolated spinal cord can generate stance and gait when animals are tested on treadmills, showing automatic adjustment to belt speed, belt direction and body weight. This motor activity may be entirely driven by afferent input. The function must be trained by repetition following the injury and this training may be functionally-specific so that animals trained to walk can walk but not stand while those trained to stand will stand well but walk badly (122). The argument for LTT has been that the patients should do walk-training and that if there is some supraspinal input, these neurons may be able to engage with the spinal circuits that generate the gait, converting it from entirely afferent-driven toward normal, with both peripheral afferent input and sensory-motor signals between the lumbar cord and the brain.

In their review, Dietz and Fouad (120) claim that LTT functional training enhances the residual plasticity. Morrison's study (34) supports this claim, showing the functional improvements that accompanied the improved motor score from LTT without electrical stimulation. Results of combining LTT with ES are equivocal. The marked improvement seen by Herman et al. (81) in one person was not seen by Field-Fote and Roach (30) in their study.

However, Table 2 shows that a positive neuroplastic effect can be produced by walking or cycling, cycling with ES being faster than LTT with ES. Studies are now needed to find out what functional recovery occurs after FES-cycling therapies, perhaps both SCS and FES. It may be that cycling therapy needs to be followed by walk-training, for the patients to experience the full benefit from the neuroplastic recovery, but this might still be quicker and less costly than LTT alone.

### Descending Drive

In clinical use, robotic LTT and cycling (passive or combined with ES) are done with little or no incentive for voluntary drive. The legs are moved by a motor, constrained by attachment to the device, and no effort is required from the patient. This is advantageous (cheaper) because it requires minimal supervision or assistance from a therapist. While the passive leg movement by the motor provides useful proprioceptive feedback, there may be little supraspinal input due to the patient's effort. Voluntary drive is however inherent in some rehabilitative interventions, such as overground walking or LTT without robotic assistance, or it can be encouraged using biofeedback. If a person with a neurologically-complete lesion becomes able to produce contractions in their otherwise paralyzed muscles in the presence of sub-threshold SCS, as has been observed in SCS studies (86, 123), the demonstration would provide a strong incentive for therapy. If FES is being applied, the muscle contractions that are the direct response to the stimulation would mask voluntarily-produced muscle activity.

It has been hypothesized that the combination of ES with descending motor commands is essential for beneficial neuroplastic change: both afferent and antidromic electrical impulses in lower motor neurons have been proposed to have a facilitatory effect on anterior horn cells, increasing their probability of firing during descending voluntary drive, resulting in an Hebbian-type learning effect (124). This is supported by observations with paired associative stimulation, which found that increases in corticomotor excitability depend on the phase during the gait cycle (125) and the reported plasticity of sensorimotor cortical structures after repetitive ES over the common peroneal nerve (97), which are dependent on concurrent voluntary drive present at the time of FES (99).

Motor training consisting of voluntary movements has been found to be more effective than passive motor training in eliciting performance improvements and cortical reorganization (126). This may be explained by voluntary effort facilitating central nervous system excitability (127) due to increased excitability of cortical motoneurons (128) and intracortical facilitation (129). Indeed, in patients with stroke, ES used with rehabilitation interventions has been reported to be more effective when combined with instructions to the patient to try to co-activate the stimulated muscles voluntarily (130, 131). The comparison between the Yaşar study (62) and our own (61) supports that idea that voluntary drive, encouraged by the virtual reality and biofeedback, improves recovery rate during FES-cycling (61).

It has also been proposed that voluntary drive does not directly facilitate cortical activity, but instead reduces inhibition at the cortical level (132). When combing FES cycling with VR, we observed an unexpected reduction in power output at the start of the VR game in some patients (i.e., when the participant was instructed to contribute voluntarily to the cycling) (61). This was principally due to co-contraction of lower limb muscles presumably due to lower limb spasms, which may have been caused by reduced levels of descending inhibition. While the addition of voluntary drive appears to be effective in achieving neuroplasticity, these observations should be taken into consideration when designing the most suitable therapy for individuals with spasticity, and the incorporation of sub-threshold tonic stimulation may be beneficial.

### Appropriate Future Therapy: Cost

LTT has clearly been shown to cause significant functional recovery in patients with AIS C and D injuries and this is maintained after the therapy (34, 133). For example, in the latter study, 45 participants did the 6-min walk test before and after at least 120 training sessions, increasing on average from 13 to 115 m. There were many accompanying improvements in autonomic function.

But what will healthcare systems pay for? In the UK, decisions about funding are based on subjective evaluation by the patients. The total value of a treatment depends on the product of the improvement in the quality of life and the number of years that it will apply. The unit is the Quality-Adjusted Life Year (QALY) and the National Health Service will usually pay about £25,000/QALY (134). The preferred measurement of Quality of Life is the SF-6D Health Utility Scale.

Lee et al. (135) used the SF-6D on 305 Australian patients with SCI. In the general Australian population, the mean score is 0.80; for paraplegics it was found to be 0.72, and for tetraplegics, 0.66. The differences between these means give some idea how much subjective improvement might be expected from treatment: a change equal to the difference between tetraplegia and paraplegia would probably be regarded as a remarkably successful therapy, yet is only a 6% improvement in quality of life. If, for example, we propose to treat people of middle age, and assume a life expectancy of 20 years, then, ignoring a discount rate, which is currently 1.5%, the QALY value would be 20 × 0.06 = 1.2. This would justify an expenditure of ~£30,000. What the actual gain in quality of life would be, will not be known until it is included in a clinical investigation.

Some of the studies cited involve 100–200 locomotor training sessions. In a recent paper, Behrman recommended at least 60, lasting 1½ h per day for successful Activity Based Therapy (7). Morrison (34) provides useful cost data from the US. Based on her figure, the cost of 120 LTT sessions, her preferred number, would be over $22k. The normal allowance from the health insurance companies in the US is 20 sessions of physiotherapy. She argues that given the improvement in walking ability and autonomic function seen in her participants, a longer course of therapy would actually save costs because of the reduced incidence of SCI complications, such as urinary tract infections and pressure sores.

This argument in favor of preventative treatment can be made in other countries and might prevail over subjective (QALY) reasoning. However, there is no doubt that LTT would take significant resourcing; Behrman et al. (7) advises: “Expanding staffing resources [and] equipment … are advantageous to successful delivery of the intervention,” implying that there will be additional estates costs also.

An alternative to LTT would be to use FES-cycling at home with some supervision from therapists. The frequency of the exercise would depend on the patient but could be done when convenient for them, and they could continue as long as the improvements justify the time taken. Changes in the motor score due to the neuroplastic response would not necessarily translate to useful functions for activities of daily living (transfers, stairs, etc.) but when the therapist can see what muscles have been restored to voluntary control, they can discuss with the patient what rehabilitation goals are realistic and might be achieved by conventional physiotherapy.

A comparison is needed between LTT, done with electrical stimulation assisted by physiotherapists, and FES-cycling done at home. It will be more complicated when neuromodulation of the posterior roots is added to the LTT, either using transcutaneous electrodes or implanted stimulators and epidural electrodes. In their single case study, Herman et al. (81) show clearly how walking speed improved when SCS was added to the LTT.

Having an implanted neuromodulator or neuroprosthesis, beside the extra cost of the device and surgery, is much more of commitment for hospital and patient: it may need adjustment from time to time, and might be used for the remainder of the patient's life. Of course, like footdrop stimulators, some patients may find that the therapeutic effect is so great that use of the device becomes unnecessary, but that does not seem to have been true for the 4 AIS A and B participants described by Angeli et al. (89).

The purpose of the implant might be to deliver a therapy for a limited period of time, after which it would be removed or simply left in place and no longer used. For devices that enable function, which otherwise would be impossible for the patient, this function might be an exercise, for example anaerobic exercise or muscle bulk maintenance, or it might allow activities of daily living. We expect that if the implant is to be successful, it should easily allow ADL and that these activities will bring the benefits of exercising the otherwise paralyzed muscles. People with SCI generally have less time than the general population for extra exercise. Facilitating use for ADL should be the goal for the therapists and implant designers.

## Conclusions

In people with chronic incomplete and discomplete SCI, recovery of leg function may be possible with afferent input to the lumbar spinal cord caused by electrical stimulation combined with repetitive training. The stimulation may be either FES or SCS, but this should be phasic, synchronized to the flexion and extension of the legs while walking or cycling, and should coincide with descending voluntary drive, to optimize neuroplastic recovery. Tonic, sub-threshold SCS appears to be effective as a long-term intervention to enable volitional movements in people with motor complete SCI (AIS A and B), at least in part by restoring spinal and descending inhibitory control, but there is currently little evidence for neuroplastic recovery.

If training is done by cycling on a stationary machine, neuroplastic change is faster if there is virtual reality and biofeedback to motivate the patient. We attribute this to the importance of supraspinal drive which may otherwise be lacking. Cycle training should be less expensive than locomotor training because it could be done at home, however, it is an established point of view that patients using a wheelchair should learn to walk, if possible, and do so by locomotor training. Therefore, future research should investigate to what extent cycle training enables patients to attain useful rehabilitation goals and improve their quality of life.

Based on the small amount of evidence available, it appears that neuroprostheses that stimulate the posterior roots will give better functional performance compared to therapy alone. These neuroprostheses solve three major problems with FES implants. However, the cost of such implants is likely to be high, at least because of the regulatory requirements (136) and establishing clinical centers with sufficient expertise and long-term commitment may be a challenge.

## Author Contributions

Both authors contributed to the article and approved the submitted version.

## Conflict of Interest

The authors declare that the research was conducted in the absence of any commercial or financial relationships that could be construed as a potential conflict of interest.
